# Nonlinear Distribution Pattern of Hibernating Bats in Caves along an Elevational Gradient in Mountain (Carpathians, Southern Poland)

**DOI:** 10.1371/journal.pone.0068066

**Published:** 2013-07-05

**Authors:** Krzysztof Piksa, Jakub Nowak, Michał Żmihorski, Wiesław Bogdanowicz

**Affiliations:** 1 Cracow Pedagogical University, Institute of Biology, Kraków, Poland; 2 Cracow Caving Club, Kraków, Poland; 3 Museum and Institute of Zoology, Polish Academy of Sciences, Warszawa, Poland; Università degli Studi di Napoli Federico II, Italy

## Abstract

**Background:**

Thermal gradients along changes in elevation in mountainous environments are reflected by different biotas. Although there have been studies of elevation variation in bat assemblages in summer, winter changes in the same gradients remain unknown.

**Methodology/Principal Findings:**

The objective of this study was to document changes in the species composition of bats hibernating in caves along a temperate elevational gradient. We studied 70 caves between from 300 m to 1,930 m altitude along a slope of the Carpathian Mountains in southern Poland. We recorded changes in bats, including species richness, abundance, altitudinal distribution and dominance during consecutive winters between 2003 and 2009. Similarity of dominance of faunal structure was assessed by using the Bray-Curtis similarity index. We used the generalised additive model and rarefaction to study the variation in species richness, and generalized additive mixed models to examine the effect of abiotic factors on the qualitative and quantitative structure of bat assemblages. During 351 surveys we recorded 13,856 hibernating bats from 15 species. Species richness peaked around mid-elevation (1,100–1,400 m a.s.l.) with richness declining at both higher and lower elevations. Based on the results of a cluster analysis, we could distinguish among four altitudinal zones that differed in species richness and dominance structure.

**Conclusions/Significance:**

This is the first study documenting changes in species richness and variation of structure of bats hibernating in caves along an elevational gradient. The most surprising and key finding is the fact that changes in the structure of assemblages of hibernating bats along the altitudinal gradient occurred in jumps, forming zones similar to those observed in the vegetation zones. Moreover, species richness and dominance structure of assemblages of hibernating bats in the mountains depended not only on location above sea level, but also on local geomorphologic conditions which strongly affected the microclimate of the caves.

## Introduction

Mountainous environments vary with altitude affecting vegetation and animal life [1]. One of the most visible effects of this impact is vegetation zones and the differentiation of the structure of animal assemblages. This phenomenon has been a subject of numerous studies of both flora [2]–[4] and fauna [5]–[8], focused on the mechanisms and patterns of species distribution [9]–[18].

Altitudinal variation in bat assemblages has been studied on nearly all continents: North America [19]–[21], South America [22]–[24], Europe [25]–[28], Asia [29]–[31] and Africa [32], [33]. However, almost all of these reports are based on the situation in summer. The situation in hibernation is not well known. There are only a few winter studies (e.g., in the USA [20], Slovakia [26] and the Czech Republic [27]). We expect that elevation effect in species distribution differs to a great extent between summer and winter as different environmental factors are important for bats during these two periods.

For temperate zone insectivorous bats winter is a critical season because of harsh climatic conditions and the attendant lack of food. There are two strategies for surviving winter, bats that migrate to warmer regions [34], [35], and those that remain and hibernate [36], [37]. Selection of appropriate roosting conditions is therefore crucial for survival of bats [37]. For most temperate bat species primary winter shelters are underground, mainly in caves or mines. The main advantages of such shelters include their thermal resistance, mechanical stability, wide range of microclimatic conditions [38] and minimization of threat from potential predators, ectoparasites and disease transmission [39]–[41].

Microclimatic requirements for hibernation differ among species of bats [42]–[47]. Some species hibernate in a wide range of *temperatures*, while others tolerate smaller variations, preferring lower or higher temperatures [47]. Taking into account the variety of microclimatic requirements of different bat species and linear decline of temperatures with altitude by an average of 0.3–0.6°C for every 100 m [48], [49], we may expect high variations in the faunal composition of hibernating bats in caves situated at different elevations.

We predicted that the altitudinal pattern of assemblages of hibernating bats would vary with altitude and species richness would decline with increasing elevation. However, origin of the caves, their geological structure, mechanism of air circulation in great subterranean systems, and microclimate of the caves situated across elevation differ considerably [50]–[54]. We expected that for bats microclimate would be more important than altitude. We put forth the hypothesis that the composition of bat assemblages occurring at different altitudes do not have to show simple linear decline with increasing elevation because microclimate and geomorphology may drive non-linear associations between bat diversity and elevation. We also assumed, as caves occur in different vegetation zones, that the type of vegetation surrounding the caves (presence and type of forest) may also have some impact on the structure of hibernating bat assemblages.

## Materials and Methods

### Study Sites

The study was conducted in the southern part of Poland in the Carpathian Mountains. The Carpathians are the greatest mountain system of Central Europe, and in Poland cover 1,600 km^2^, with the altitudinal range of ca. 190 m to 2,499 m. Despite being lower than the Alps, the Carpathians have similar landscapes and characteristics (e.g., climatic and vegetation belts) typical of higher mountain chains [55]. The relatively small study area, the large number of caves covering almost the entire altitudinal spectrum, and the relatively easy and safe access to the farthest and highest underground sites (a few hours to reach the openings of all caves in winter) present an excellent opportunity for this type of study.

**Figure 1:**
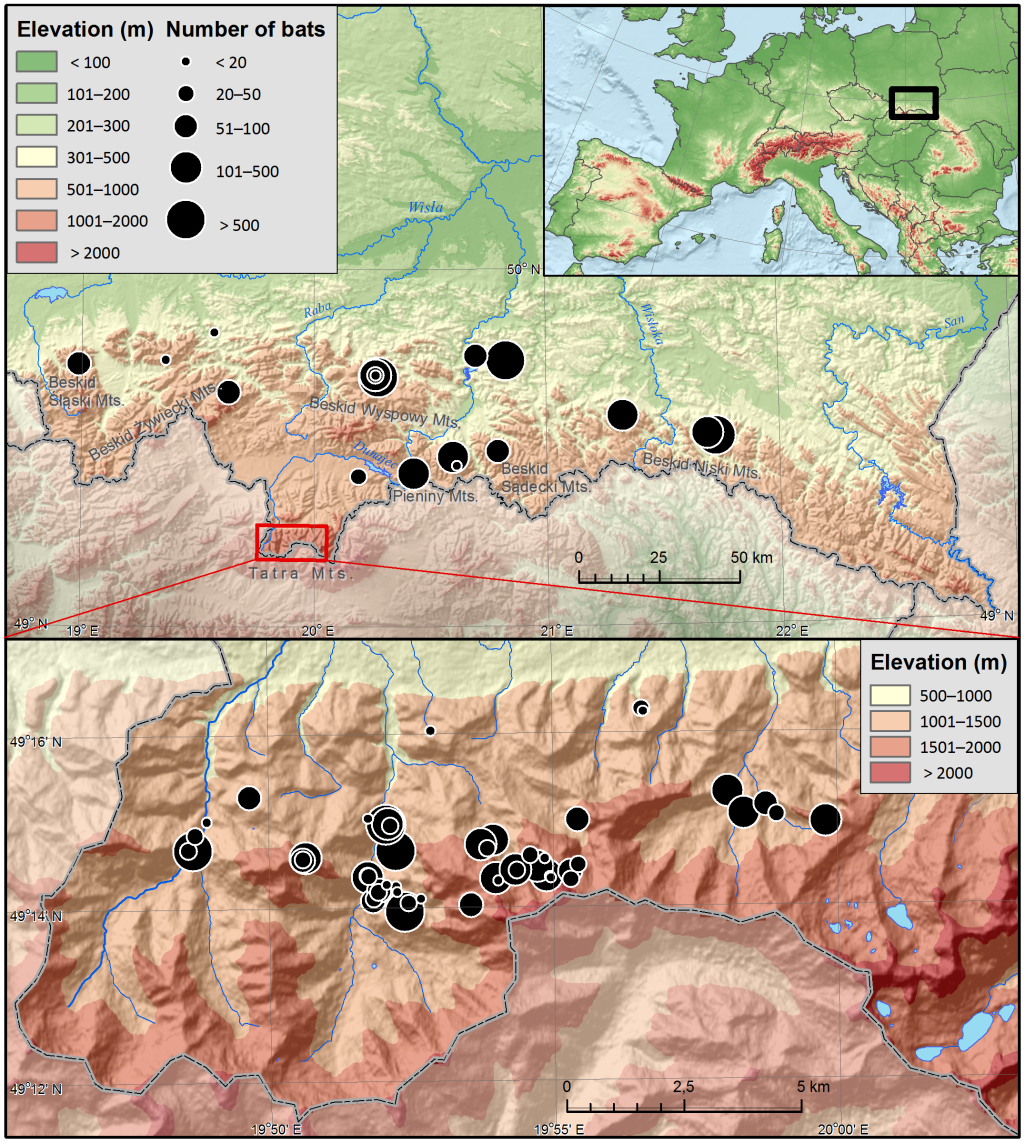
Location of Poland in Europe (top right), study area (70 caves) in the southern part of the country (middle), and location of surveyed caves in the Tatra Mts. (bottom). The size of circles represents the sample size.

We selected 70 caves between 300 and 1,930 m a.s.l. (1,630 m gradient) (Figure 1). The caves are located in five of the six Carpathian climate zones (from moderate warm to moderately cold) and differ in size, origin, and type of air circulation, and therefore in their microclimatic conditions as well. Caves located below ca. 1,000 m a.s.l. (with two exceptions) are typical non-karstic caves, while the remaining ones are of karstic type. Nineteen caves are situated in the Outer Carpathians (Beskids), two in the Pieniny Mountains and the remaining in the Tatra Mountains (Figure 1). Most caves above the upper forest range limit of 1,500 m a.s.l. are typical alpine, vertical caves.

Field surveys were carried out during consecutive hibernation periods from 2003/2004 to 2008/2009. Most of the caves were surveyed during the mid winter (January and February). Due to difficulties in survey during the high winter season, i.e. changing weather, difficulties reaching the cave entrances (many times impossible by thick snow cover burrying the entrances), and the risk of avalanches, several of the caves above the upper forest range and a few below that, were surveyed early (November and December) or late during the hibernation period (even in April). Many caves are also very large and technically difficult to monitor, requiring up to 48 hours of work, and that has also hampered the work. In some cases, we could not perform regular annual censuses.

### Species Identification

Bats were identified based on differences in morphology. Due to the intrinsic difficulty in differentiating bats from the *M. mystacinus* group, i.e., *Myotis mystacinus* s.s., *M. brandtii*, and *M. alcathoe*, during winter surveys we refer to them as *M. mystacinus* s.l. and report the census data together. The specific assessment of species from this group was based on individuals found dead in the Carpathian caves during the winter and post-hibernation periods, and analyses of photographs and genetic profiles.

### Cave Microclimate

We used four principal parameters: average cave air temperature, temperature range and relative humidity and its range to characterize the microclimates of the caves. Air temperature was measured outside the cave, at its opening, and at 4–10 locations along the main course of the cavern. The temperature range was determined as the difference between the highest and the lowest temperatures recorded in the vicinity of a bat in that cave. Air temperatures were recorded by AM 9112 thermometer (KFAP, Poland) and/or thermo-hygrometer RhT-15 (Termoprodukt, Poland). Humidity was measured using a RhT-15 thermo-hygrometer and/or an Assman psychrometer.

### Analyses

Resemblance of wintering bat assemblages between the sites was analyzed using a clustering analysis. To avoid sampling biases toward one area of the mountain and to standardize sample sizes we selected 33 (Table S1) of the 70 surveyed caves for this purpose, i.e., 1–3 randomly selected caves (if available) per every 100 meters of the altitudinal gradient. We did not include unidentified bats in the analysis. Similarity of dominance of structures of faunas was assessed by matrices calculated using the Bray-Curtis similarity index [56]. We used this index because it has many properties amenable to ecological data, including independence from both the scale of measurement and joint absences [57], [58]. Analyses were performed using Statistica v. 9.0 package (Statsoft Inc., Tulsa, USA).

Next, we used rarefaction curves to compare species richness of bat assemblages in the four selected elevation zones. The rarefaction curves present expected cumulative number of species as a function of sampling effort, which can be expressed with number of sampled individuals (individual-based rarefaction) or number of samples taken into account (sample-based rarefaction) [59]. We used both approaches with the help of EstimateS 800 software [60]. We drew the curves for each elevation zone independently as well as for the pooled material. Additionally, we pooled together the two highest zones as numbers of bats recorded in these two zones are low. In this analysis all the individuals determined as *Myotis mystacinus* s.l. were treated as one species.

To study variation in species richness of bats in the examined caves, we conducted two analyses – first, we explored the effect of elevation using the full data set (n = 70), and second, we included the microclimate and geomorphology using the smaller set of caves (30 out of the 33 caves used in ecological analyses; three caves were omitted due to failure of measuring instruments and the lack of microclimatic data). As a first step, we applied additive modelling as one may expect nonlinear patterns. More specifically, we used a generalised additive model (GAM) with the Poisson error distribution and log link, where the number of species in each cave was the response variable and the elevation was the explanatory variable. The elevation was modelled as a covariate fitted with penalized cubic regression splines. Modelling was performed with the help of “mgcv” package [61] in the R program v. 2.14 [62]. In the case of variability of abiotic characteristics of the caves used by bats for wintering, we applied the same procedure with the elevation effect fitted with the spline and the type of cave (karstic vs non-karstic) as a fixed categorical factor.

Next, we used generalized additive mixed models (GAMM) to study the effect of microclimate (covariates: temperature, its amplitude and relative humidity), elevation (covariate), type of cave (fixed factor: karstic vs non-karstic) and type of vegetation surrounding the caves (fixed factor: deciduous forest, mixed forest, coniferous forest and open) on the number of bat individuals and the number of bat species in a given cave and season (two independent modelling procedures were performed). As the number of explanatory variables was too high for a relatively restricted dataset we decided to omit the amplitude of humidity, which seems to be of least importance and shows rather small variation across caves and seasons. A season was used in the models as a random factor whereas the effects of continuous variables were fitted with penalized cubic regression splines.

### Ethics Statement

All procedures were carried out under licenses from Ministry of Environment and from National Parks in Poland. Ministry of Environment specifically approved our study. Data used in calculations are available upon request from the corresponding author.

## Results

### Cave Microclimate

**Figure 2:**
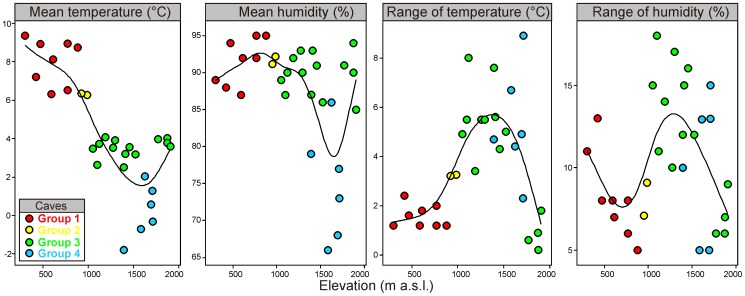
Variability of four abiotic characteristics of 30 caves in the Polish Carpathians used by bats for wintering as a function of elevation fitted with splines (solid lines). The four groups of bats obtained by cluster analysis (see Figure 6) are presented with different colours.

The four abiotic features we measured in caves, changed significantly with the increasing elevation and the relationship between these parameters and elevation was not linear (Figure 2). The lowest values of both the mean temperature and the mean humidity were recorded in caves situated between 1,500–1,700 m a.s.l. The greatest ranges of temperature and humidity were observed between 1,100 and 1,500 m a.s.l. Two out of the four microclimate characteristics differed between two types of caves (independently on the elevation). Average temperature was by 2.4 degrees higher (SE of this estimation = 1.06) in non-karstic caves as compared to karstic ones (Linear model, t-value = 2.29, p = 0.031). The range of humidity also appeared to depend on the cave type, being lower by 5 percentage points in non-karstic than in karstic caves (Linear model, t-value = 2.14, p = 0.042). The range of temperature and humidity itself did not depend on the cave type (p>0.2 in both cases).

### Number of Bats and Species Compositions

**Figure 3:**
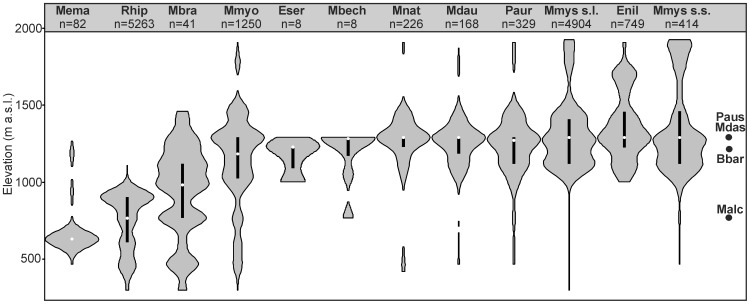
Distribution pattern of bats wintering in caves in the Polish Carpathians along the elevation gradient expressed with kernel density estimators and boxplots. Total abundance of each species is given below its abbreviated name: Mema – *Myotis emarginatus*, Rhip – *Rhinolophus hipposideros*, Mbra – *M. brandtii*, Mmyo – *M. myotis*, Eser – *Eptesicus serotinus*, Mbech – *M. bechsteinii*, Mnat – *M. nattereri*, Mdau – *M. daubentonii*, Paur – *Plecotus auritus*, Mmys s.l. – *M. mystacinus* sensu lato, Enil – *E. nilssonii*, Mmys s.s. – *M. mystacinus* sensu stricto, Malc – *M. alcathoe*, Bbar – *Barbastella barbastellus*, Mdas – *M. dasycneme*, Paus – *P. austriacus*.

During 351 surveys in 70 caves we recorded 13,856 hibernating bats from 15 species (Table 1). The most numerous species were *Rh. hipposideros* (38.0% of the total) and *M. mystacinus* s.l. (35.4%). Relatively large numbers of *M. myotis* (9.0%) and *E. nilssonii* (5.4%) were also recorded. The remaining species were observed in much small numbers. A large group of bats could not be identified to species or group of species (862 individuals). Taxa differed from each other in their altitudinal range and abundance (Figure 3).

**Table 1:** Number of hibernating bats in caves in the Polish Carpathians.

	Season						
Species	2003/2004	2004/2005	2005/2006	2006/2007	2007/2008	2008/2009	Total
*Rhinolophus hipposideros*	630	848	920	951	1033	881	5,263
*Myotis myotis*	163	199	202	219	267	200	1,250
*M. bechsteinii*		1		1	2	4	8
*M. nattereri*	38	36	33	36	46	37	226
*M. mystacinus* s.l.	431	686	680	922	1229	956	4,904
*M. daubentonii*	18	27	30	19	42	32	168
*M. dasycneme*						1	1
*M. emarginatus*	1	10	14	15	20	22	82
*Eptesicus nilssonii*	92	131	137	156	119	114	749
*E. serotinus*		1	1	1	2	3	8
*Plecotus auritus*	39	49	40	56	73	72	329
*P. austriacus*					1		1
*Barbastella barbastellus*	1	1	1	1			4
Unidentified	98	93	156	161	189	166	863
Total (number of surveys)	1,511 (58)	2,082 (58)	2,214 (62)	2,538 (60)	3,023 (64)	2,488 (50)	13,856 (351)

Number of surveys (winter censuses) is given in parentheses.

### Species Richness and Diversity

**Figure 4:**
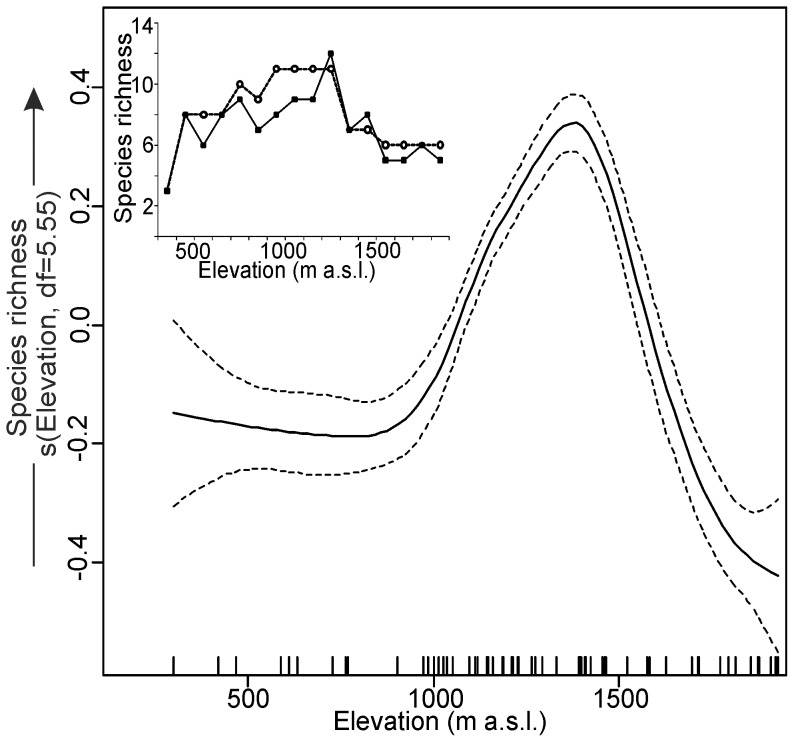
Spline fit (solid line) with 95% confidence interval (dashed lines) of the variability in the species richness of bat assemblages wintering in 70 caves in the Polish Carpathians as a function of elevation (GAM: species richness∼intercept+s(elevation)). In the inner subplot empirical (solid line and black squares, number of recorded species on each 100-m vertical bands) and interpolated (dashed line and open circles, species was recorded at range between the highest and the lowest records) species richness are presented.

**Figure 5:**
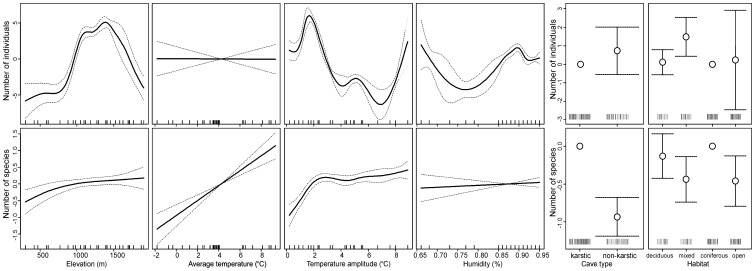
Spline fits (solid lines) with 95% confidence intervals (dashed lines) of the variability in species richness and abundance of bats wintering in 30 caves in the Polish Carpathians as a function of elevation, average temperature, amplitude of temperature, humidity, type of cave and type of vegetation (GAMM: species richness/abundance∼intercept+s(elevation)+s(average temperature)+s(amplitude of temperature)+s(humidity)+type of cave+vegetation+random(season)). In both models the season was included as a random categorical factor.

The GAM model, using elevation as explanatory variable, showed a significant nonlinear pattern in the species richness of bat assemblages along the altitudinal gradient (Figure 4). The effect of elevation fitted with the spline was highly significant (df = 5.55, p<0.001), suggesting the highest richness around 1,400 m a.s.l. The adjusted determination coefficient for the model denoted 32%. Interpolated and empirical data showed similar pattern – the mid-elevational peak in species richness at 1,100–1,400 m a.s.l. (Figure 4, inner subplot). The GAMM models showed a difference in species numbers between the two cave types, with species numbers being reduced in non-karstic caves. No such differences were observed in bat abundance (Table 2, Figure 5). Different patterns were observed for abundance and species richness in assemblages of bat hibernating in caves surrounded by different vegetation types. On average bat abundance reached the highest value in mixed forest habitats whereas species richness peaked in coniferous forests (Table 2, Figure 5). The majority of covariates appeared to significantly affect abundance and species richness. Non-linear relationships predominated, showing that linear modelling in this situation may lead to biased conclusions. However for one variable – average temperature – the observed relationship was linear (Table 2, Figure 5). The effect of elevation suggests that the highest abundance of bats may be expected at moderate altitudes. The effect of temperature is positive for species richness and has no influence on bat abundance. The effect of humidity on bat abundance is far from linear, and seems to be rather difficult to interpret.

**Table 2:** Summary of generalised additive mixed models (GAMM) explaining the number of bat individuals and the number of bat species in 30 caves in the Polish Carpathians between 2003 and 2009 on the basis of microclimate (average temperature, its amplitude and humidity), elevation and type of cave (karstic vs non-karstic) and vegetation type (deciduous forest, mixed forest, coniferous forest, open).

Dependent variable: number of individuals			
Fixed effect	Estimate (SE)	t-value	p-value
Intercept	3.009 (0.505)	5.962	<0.001
Cave type = non-karstic	0.726 (0.639)	1.137	0.259
Cave type = karstic	0		
Habitat = open	0.226 (1.345)	0.168	0.867
Habitat = forest mixed	1.476 (0.526)	2.805	0.006
Habitat = deciduous	0.112 (0.339)	0.329	0.743
Habitat = coniferous	0		
**Smooth terms**	**Estim. df**	**F-value**	**p-value**
Average temperature	1.00	0.002	0.967
Elevation	7.68	48.022	<0.001
Humidity	6.44	16.838	<0.001
Temperature amplitude	7.94	65.710	<0.001
**Dependent variable: number of species**			
**Fixed effect**	**Estimate (SE)**	**t-value**	**p-value**
Intercept	1.625 (0.072)	22.476	<0.001
Cave type = non-karstic	−0.935 (0.128)	7.298	<0.001
Cave type = karstic	0		
Habitat = open	−0.460 (0.167)	2.756	0.007
Habitat = forest mixed	−0.437 (0.151)	2.899	0.004
Habitat = deciduous	−0.129 (0.147)	0.877	0.382
Habitat = coniferous	0		
**Smooth terms**	**Estim. df**	**F-value**	**p-value**
Average temperature	1.00	33.924	<0.001
Elevation	2.31	4.259	0.013
Humidity	1.00	0.363	0.548
Temperature amplitude	5.21	10.194	<0.001

In the examined models, the season was included as a random categorical factor but its effect is not presented.

### Resemblance between Bat Assemblages

**Figure 6:**
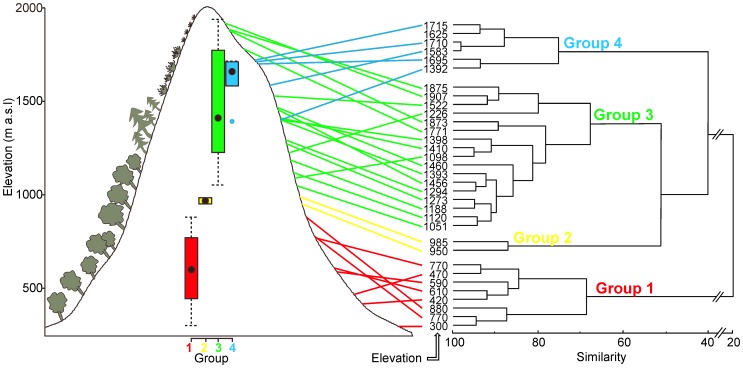
Cluster analysis of bat assemblages wintering in 33 caves in the Polish Carpathians. Four groups were distinguished and marked in different colours; the altitudinal position of openings of caves is projected on the slope and expressed with a boxplot. Four main vegetation zones are drawn on the left slope.

The cluster analysis of bat assemblages in 33 caves divided the caves into four groups (Figure 6). All caves situated up to ca. of 900 m a.s.l. were classified as group no. 1, two sites situated at 950 and 985 m altitude were classified to group no. 2, 13 caves between of ca. 1,000 and 1,520 m a.s.l. and all above altitude of 1,771 m were classified to group no. 3 and all caves between 1,576 and 1,715 m altitude and one cave at 1,342 m a.s.l. to group no. 4. In other words, group 4 was located within the range of group 3 (Figure 6).

**Figure 7:**
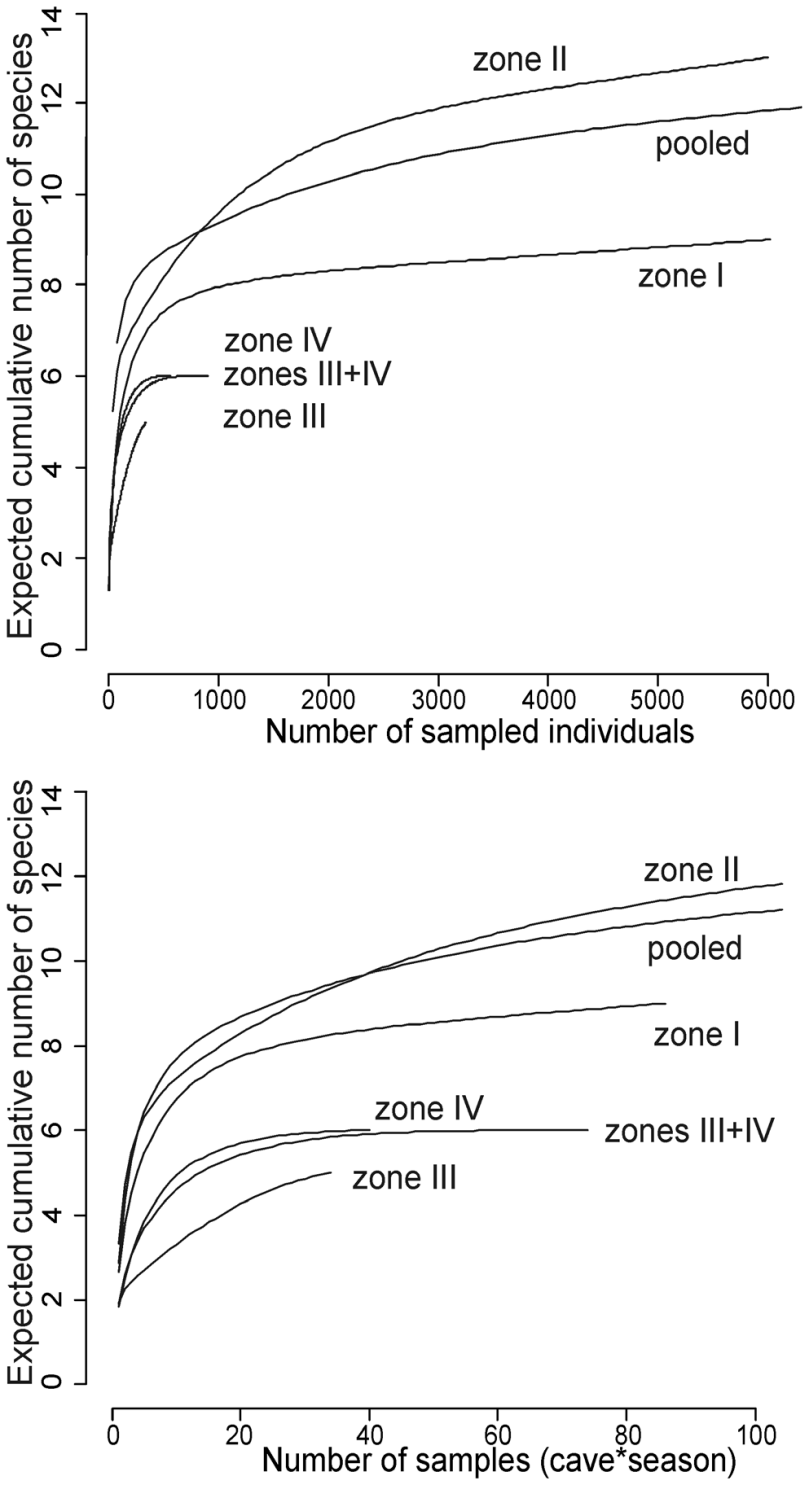
Expected cumulative number of bat species as a function of sampling effort: number of sampled individuals (upper panel) and number of caves controlled in one season (lower panel).

Rarefied diversity of bat assemblages showed high variability in species richness between the four elevation zones. In both approaches zone II was the most diverse and the only zone more diverse than the pooled data. The two highest zones (III and IV) were the least diverse (Figure 7).

### Pattern of Elevational Distribution

Altitudinal distribution of groups derived from cluster analysis (Figure 6) and elevation ranges of bat species (Figure 3) allow us to recognize four zones of caves in the Carpathian Mountains each with a distinctly different ecological index. The upper and lower limits of each zone were defined as the highest and lowest locations of the openings of the caves from the group defined by cluster analysis (see Figure 6).

Zone I. Up to about 1,000 m a.s.l, this zone includes caves from groups 1 and 2 (Figure 6) in which we found 10 species. The most prevalent was thermophilous *Rh. hipposideros*, accounted for 91.5% of all bats. The number of remaining taxa, excluding *M. myotis*, which comprised 5.5% of all bats, was very low (174 individuals). Air temperatures and humidity of the caves were relatively high and the ranges of these parameters were low (Figure 2).

Zone II. The caves of group 3 between c.a. 1,000–1,520 m showed the greatest richness of species (14 species). The most prevalent was *M. mystacinus* s.l. (66.5%), as well as *M. myotis* and *E. nilssonii* (13.9% and 9.0%, respectively). The ranges of air temperature and humidity of the most caves were much wider than in the previous zone (Figure 2).

Zone III. From 1,520 m and to below 1,715 m, were the caves of group 4 characterised by a drastic decrease in abundance and species richness (4 species). The most prevalent (66.0%) was psychrophilous *E. nilssonii*, while *M. mystacinus* s.l. was often observed as well (33.2%). The microclimate of these caves was the most harsh (in some of them the mean air temperature was below 0°C and relative humidity was reaching the lowest level; see Figure 2).

Zone IV. The area above 1,715 m includes caves from group 3 and we recorded six species there; *M. mystacinus* s.l. being the most prevalent (88.5%), *E. nilssonii* – the most numerous species in the previous zone – was almost absent. The humidity and air temperature of the caves were higher than in the previous zone and the microclimate was more stable (Figure 2).

We identified 456 individuals to species belonging to the *M. mystacinus* group (414 *M. mystacinus*, 41 *M. brandtii* and 1 *M. alcathoe*). Two species (*M. mystacinus* and *M. brandtii*) differed from each other in the altitudinal range (Figure 3) and abundance between the distinguished zones (*χ2* = 136.6, df = 2, p<0.001; data from zones III and IV were pooled). *M. mystacinus* dominated in zone II (314 individuals vs 18 individuals of *M. brandtii*) and was the only species recorded in zones III and IV (19 and 66 individuals, respectively). In contrast, in zone I *M. mystacinus* was less numerous than *M. brandtii* (15 vs 23 individuals, respectively).

## Discussion

We documented the presence of 15 of the 25 bat species found in Poland [63]–[65], including Mediterranean elements (e.g., *Rh. hipposideros*, *M. myotis* and *M. emarginatus*), azonal euconstant species (*M. mystacinus*), arboreal true Eupalearctic species (*P. auritus* and *M. daubentonii*), arboreal West-Palearctic species (*M. nattereri*, *M. brandtii*), and boreal-mountainous species (*E. nilssonii*) (sensu [66]). Different zoogeographic characteristics of these bats were reflected in their varying microclimatic requirements during hibernation, which ranged from typically thermophilous *Rh. hipposideros* to typically psychrophilous *E. nilssonii* [42], [43], [46], [47].

The sympatric and repeated occurrence of bats from the *M. mystacinus* morphogroup (*M. mystacinus*, *M. brandtii* and *M. alcathoe*) is also worth mentioning. Despite morphological similarities, all three species showed different altitudinal preferences in winter: *M. brandtii* occurs at lower elevations, whereas *M. mystacinus* s.s. seems to be more flexible and cold-hardy, and hence it shows a greater range of vertical distribution and overwhelming dominance in the caves at higher elevations. Our observations and others in southern Poland [67] suggest that *M. alcathoe,* similar to *M. brandtii*, prefers lower elevations during hibernation. Comparable preferences were observed during swarming activity of these bats in late summer and autumn in the Carpathians [68], [69].

We found that the species richness of faunas of hibernating bats was not evenly distributed with altitude, reaching the highest values approximately in the middle of the examined altitudinal gradient. Such mid-elevation peaks in bat richness have also been recorded in the mountains during summer, e.g., in the Southern Alps, France [25], Sierra Nevada [21], White and Inyo Mountains [20], and Henry Mountains, USA [13]. This reflects that some species migrate up the mountains to hibernacula, while others come down. Evidently, this “humped” species richness pattern of bats recorded in the Polish Carpathians based on winter data does not differ from that observed during activity period of these animals in other mountains. This type of altitudinal pattern in the bat species richness is characteristic for both temperate mountains and vespertilionids bats [13].

We also noted significant differences in the number of species and the overall number of individuals between assemblages of bats hibernating in caves surrounded by different vegetation types. This finding suggests that the type of vegetation may have some impact on the structure of hibernating bat assemblages. Nevertheless, as shown in this study as well as other studies [44]–[46], [70] the main factor determining the species richness of hibernating bat assemblages is the microclimate of winter roosts. In the Carpathians the highest species richness and the highest bat abundance were observed at mid-elevation in the caves located in areas dominated by coniferous and partly mixed forests. At this altitudinal range there are caves with the greatest diversity of microclimatic conditions recorded. In contrast, in the areas covered by deciduous forests (up to 1,000 m a.s.l.) and open (devoid of trees) at high altitudes (above 1,500 m a.s.l.) there are caves either with the lowest range of microclimatic conditions or with the most severe conditions – hence the low species richness of bat assemblages. In light of the above facts, the relationship between abundance/species richness and the type of vegetation surrounding the caves appears to ultimately be driven by local climate.

Elevational richness patterns depend on the organism studied, and possible causes for changes in diversity along the altitudinal gradient may be species-specific: either climatic, spatial, biotic and/or historical (invoking processes occurring across evolutionary timescales) [71]. A global meta-analysis of species richness of bats from the Old and New World mountains showed that elevational richness patterns observed in bats during summer were related to local climatic gradients. Species richness was highest where both temperature and water availability were high, and declined as temperature and water availability decreased [13]. Similarly, in the bat assemblages hibernating in the caves of the Carpathian Mountains, the most important factor modeling the species richness (as well as their group structure) is of abiotic character, i.e. microclimatic conditions of the caves: the range of temperature and relative humidity. The largest difference in these conditions is observed between the caves and cave areas at mid-elevation, hence the intermediate peak in species richness. Climatic conditions are evidently less diverse at hibernacula located at higher or lower altitudes, contributing to lower species richness.

This is the first study that documents not only changes in the species richness and zonation pattern of groups of wintering bats, but also one of very few studies describing variation in the bat fauna during winter along the altitudinal gradient in the mountains [20], [26], [27]. In our study, one of the most surprising discoveries is the fact that changes in the structure of assemblages of hibernating bats along the altitudinal gradient do not occur gradually, but in jumps, i.e. similar to those observed in vegetation zones [72]. Also, contrary to our expectations, the most severe microclimatic conditions are not observed in caves located at highest elevations, and the most cold tolerant species, such as *E. nilssonii*, are not numerous at highest elevations either.

So the question arises which factors cause variations in the structures of hibernating bat assemblages between the distinguished zones on one hand, and their unification within them on the other. To explain this phenomenon, it is necessary to look at the genesis of caves in the studied area. Those in zone I are non-karst forms, developed primarily due to gravitational movements of rock masses in mountain slopes. They generally represent two types: crevice-type formed owing to the process of slow and limited widening of joints preceding the landslide formation, and talus-type voids among large rock blocks of landslide colluvia [53]. One of the consequences of their formation is that entrances and other openings in the majority of these caves are located in their upper parts. Such a structure, relative to the opening and underground caverns, means that the air warmed in the rock fissures flows out through the cave openings – a typical chimney effect [51]. Therefore, such caves are characterised by stable microclimatic conditions, small temperature gradient and relatively high temperatures resulting from their low altitude location. Such conditions favour thermophilous *Rh. hipposideros*, which prefers warmer roosts for hibernation [42], [43]. Eurythermal *M. myotis*, with a slightly greater range of thermopreferendum [42], [43], [47], is also relatively abundant in such hibernacula. The second cave zone is located at mid-elevation and has the highest species richness and the greatest diversity of the structure of bat assemblages. High values of these variables are linked with the high heterogeneity of microclimatic conditions, providing wintering roosts with the greatest microclimatic variation. Most caves in the two highlighted zones are typical “alpine” caves located in a relatively small area in the sub-summit and upper sections of the Tatra Mountains. They probably represent one great system of coupled air corridors and gaps. This part of an underground zone is known as the trogal. In such systems the caves’ microclimate is frequently modelled by the chimney effect [54], i.e. the situation when warm, lighter air heated in the orogenic belt flows through the openings of caves situated higher up, whereas cold air is sucked through the holes and fissures of the system lying below. Therefore lower lying caves [in the case of this study the caves in zone III] have much harsher microclimate than those located at higher elevations. It is in this zone that most of the ice caves in the Tatras are located [52]. Very harsh microclimatic conditions also limit the number of bat species and lead to a dramatic decrease in their numbers. The dominant species is the extremely psychrophilous *E. nilssonii* [43], [46], [47]. Eurythermal *M. mystacinus* s.s. is also relatively numerous. Compared to the previous zone, microclimatic conditions in the highest lying caves (zone IV in the present study), are completely different. Inflowing cold air, which causes cooling in low-lying caves, circulates through the system of crevices and empty spaces in the rock trogal and is gradually heated and saturated with water vapour. Microclimatic conditions prevailing here, in contrast to the previous zone, are stable, and caves are characterised by a small temperature gradient and high relative humidity. The consequence of this is a complete reshaping of the cluster structure of hibernating bats. The highest dominance and frequency indices are recorded for *M. mystacinus* s.s., whereas *E. nilssonii* is very rare. There are also some species present within this zone that prefer higher temperatures which were absent in the previous zone, such as *M. myotis*, *P. auritus* and *M. nattereri* [43], [47].

Our research shows that aggregations of wintering bats and the temperature regime of caves in the mountains depend not only on their location above sea level, but also on local geomorphologic conditions which modelled microclimate of the caves. This raises the question whether changes in bat aggregations along the altitudinal gradient discovered in the Polish Carpathians may also be observed in other mountainous regions around the world. Because temperature is generally negatively correlated with increasing altitude, the chimney effect discussed herein is one of the most crucial factors for modelling cave microclimates important to specific species of bats. Nevertheless, such observations are likely to vary given different geographic areas because data collected along altitudinal gradients will reflect the combined effect of regional peculiarities and general altitude phenomena [49].

## Supporting Information

Table S1 **Measurements of the 33 surveyed caves and number of hibernating bats.** Abbreviations: Rhip – *Rhinolophus hipposideros*, Mmyo – *Myotis myotis*, Mmys s.l. – *M. mystacinus* sensu lato, Enil – *Eptesicus nilssonii*, Paur – *Plecotus auritus*, Unident. – unidentified.
(DOC)
