# Supplementary material for: Nonlinear Distribution Pattern of Hibernating Bats in Caves along an Elevational Gradient in Mountain (Carpathians, Southern Poland)
Source: PLoS One. 2013 Jul 5;8(7):e68066. doi: 10.1371/journal.pone.0068066 (PMC3702566; doi:10.1371/journal.pone.0068066)
Supplement: Table S1 — Measurements of the 33 surveyed caves and number of hibernating bats. Abbreviations: Rhip – Rhinolophus hipposideros, Mmyo – Myotis myotis, Mmys s.l. – M. mystacinus sensu lato, Enil – Eptesicus nilssonii, Paur – Plecotus auritus, Unident. – unidentified. (DOC) [file pone.0068066.s001.doc]

Table S1. Measurements of the 33 surveyed caves and number of hibernating bats. Abbreviations: Rhip — *Rhinolophus hipposideros*, Mmyo — *Myotis myotis*, Mmys s.l. — *M. mystacinus* sensu lato, Enil — *Eptesicus nilssonii*, Paur — *Plecotus auritus*, Unident. – unidentified.

|  | **Cave characteristics** | | | **Number of bats** | | | | | | | |  |
| --- | --- | --- | --- | --- | --- | --- | --- | --- | --- | --- | --- | --- |
| **Cave Name** | **Altitude (m)** | **Length (m)** | **Denivelation depth (m)** | **Rhip** | **Mmyo** | **Mmys s.l.** | **Enil** | **Paur** | **Others** | **Unident.** | **Total** | **Number of bat species** |
| Szkieletowa | 300 | 110 | 19 | 96 | 1 | 1 | 0 | 0 | 0 | 0 | 98 | 3 |
| Drwali (Słowiańska) | 420 | 564 | 28.3 | 254 | 24 | 0 | 0 | 0 | 1 | 0 | 279 | 3 |
| Diabla Dziura w Bukowcu | 470 | 365 | 42.5 | 658 | 48 | 6 | 0 | 5 | 10 | 1 | 728 | 8 |
| W Ociemnem | 590 | 196 | 47.5 | 218 | 38 | 0 | 0 | 1 | 7 | 0 | 264 | 5 |
| Gdzie Grotołaz Wpadł | 610 | 75 | 14.3 | 556 | 16 | 2 | 0 | 1 | 4 | 0 | 579 | 7 |
| Czarci Dół | 770 | 140 | 14 | 385 | 2 | 3 | 0 | 0 | 1 | 0 | 391 | 5 |
| Mroczna | 770 | 198 | 15.5 | 407 | 48 | 2 | 0 | 4 | 1 | 0 | 462 | 6 |
| Zbojecka w Łopieniu | 880 | 433 | 20.3 | 2,551 | 10 | 2 | 0 | 1 | 3 | 0 | 2567 | 7 |
| Trzy Kopce* | 950 | 1,244 | 32.6 | 17 | 25 | 10 | 0 | 2 | 3 | 1 | 58 | 7 |
| Niedźwiedzia | 985 | 340 | 20 | 5 | 68 | 6 | 0 | 13 | 2 | 0 | 94 | 7 |
| Szczelina Chochołowska | 1,051 | 2,320 | 60 | 0 | 143 | 326 | 18 | 11 | 18 | 27 | 543 | 8 |
| Mylna | 1,098 | 1,630 | 46 | 0 | 9 | 217 | 48 | 10 | 10 | 38 | 332 | 7 |
| Zimna* | 1,120 | 5,335 | 176 | 0 | 88 | 773 | 35 | 31 | 27 | 54 | 1,008 | 7 |
| Naciekowa | 1,188 | 1,210 | 97 | 0 | 68 | 274 | 15 | 21 | 32 | 30 | 440 | 8 |
| Za Smrekiem | 1,226 | 80 | 10 | 0 | 1 | 27 | 17 | 2 | 0 | 2 | 49 | 4 |
| Miętusia* | 1,273 | 10,780 | 305 | 0 | 27 | 171 | 4 | 16 | 16 | 25 | 259 | 7 |
| Czarna | 1,294 | 6,740 | 303.5 | 0 | 279 | 932 | 176 | 85 | 178 | 268 | 1,918 | 11 |
| Piwnica Miętusia | 1,392 | 196 | 16 | 0 | 0 | 2 | 20 | 0 | 0 | 0 | 22 | 2 |
| Miętusia Wyżnia* | 1,393 | 776 | 145 | 0 | 10 | 178 | 15 | 16 | 15 | 2 | 236 | 7 |
| Śpiących Rycerzy | 1,398 | 270 | 46 | 0 | 2 | 38 | 10 | 1 | 3 | 26 | 80 | 7 |
| Psia | 1,410 | 917 | 62 | 0 | 15 | 437 | 42 | 11 | 21 | 13 | 539 | 7 |
| Bandzioch Kominiarski* | 1,456 | 9,550 | 562 | 0 | 68 | 252 | 33 | 19 | 18 | 24 | 414 | 7 |
| Magurska | 1,460 | 1,285 | 59 | 0 | 44 | 42 | 10 | 6 | 5 | 4 | 111 | 7 |
| Nad Dachem* | 1,522 | 300 | 50 | 0 | 1 | 99 | 2 | 1 | 1 | 0 | 104 | 5 |
| Przy Przechodzie | 1,583 | 220 | 28 | 0 | 0 | 7 | 23 | 0 | 0 | 0 | 30 | 2 |
| Za Płytą | 1,625 | 62 | 27 | 0 | 0 | 17 | 13 | 0 | 0 | 0 | 30 | 2 |
| Suchy Biwak | 1,695 | 70 | 18 | 0 | 0 | 1 | 21 | 0 | 0 | 0 | 22 | 2 |
| Mechata | 1,710 | 66 | 18 | 0 | 0 | 22 | 57 | 0 | 0 | 0 | 79 | 2 |
| Lodowa w Ciemniaku | 1,715 | 390 | 42 | 0 | 0 | 23 | 30 | 0 | 1 | 4 | 58 | 3 |
| Marmurowa* | 1,771 | 681 | 150.5 | 0 | 4 | 98 | 0 | 3 | 2 | 61 | 168 | 4 |
| Małołącka | 1,873 | 258 | 166 | 0 | 1 | 18 | 0 | 1 | 2 | 6 | 28 | 5 |
| Nad Kotliny* | 1,875 | 300 | 120 | 0 | 0 | 41 | 1 | 1 | 0 | 24 | 67 | 3 |
| Wielka Litworowa* | 1,907 | 1,000 | 200 | 0 | 2 | 211 | 10 | 3 | 2 | 106 | 334 | 6 |

*caves only partially surveyed
